# Nano-Technological Approaches for Targeting Kidney Diseases With Focus on Diabetic Nephropathy: Recent Progress, and Future Perspectives

**DOI:** 10.3389/fbioe.2022.870049

**Published:** 2022-05-13

**Authors:** Bo Lin, Ying-Yu Ma, Jun-Wei Wang

**Affiliations:** ^1^ Urology & Nephrology Center, Department of Nephrology, Zhejiang Provincial People’s Hospital (Affiliated People’s Hospital, Hangzhou Medical College), Hangzhou, China; ^2^ Key Laboratory of Gastroenterology of Zhejiang Province, Zhejiang Provincial People’s Hospital (Affiliated People’s Hospital, Hangzhou Medical College), Hangzhou, China; ^3^ Clinical Research Institute, Zhejiang Provincial People’s Hospital (Affiliated People’s Hospital, Hangzhou Medical College), Hangzhou, China; ^4^ Emergency Department, Tiantai People’s Hospital of Zhejiang Province (Tiantai Branch of Zhejiang People’s Hospital), Taizhou, China

**Keywords:** diabetic nephropathy, nanotechnology, targeted delivery, kidney disease, nanoparticles

## Abstract

Diabetic nephropathy (DN) is the leading cause of end-stage renal disease worldwide. With the rising prevalence of diabetes, the occurrence of DN is likely to hit pandemic proportions. The current treatment strategies employed for DN focus on the management of blood pressure, glycemia, and cholesterol while neglecting DN’s molecular progression mechanism. For many theranostic uses, nano-technological techniques have evolved in biomedical studies. Several nanotechnologically based theranostics have been devised that can be tagged with targeting moieties for both drug administration and/or imaging systems and are being studied to identify various clinical conditions. The molecular mechanisms involved in DN are discussed in this review to assist in understanding its onset and progression pattern. We have also discussed emerging strategies for establishing a nanomedicine-based platform for DN-targeted drug delivery to increase drug’s efficacy and safety, as well as their reported applications.

## 1 Introduction

Diabetic nephropathy (DN), a frequent consequence of diabetes, has become a hazard to human health as diabetes prevalence has increased globally during the previous few decades (Kakitapalli et al., 2020). Diabetes affected more than 400 million people worldwide in 2014, and the World Health Organization predicts that diabetes is expected to become the seventh greatest leading cause of mortality by 2030 (WHO, 2016). In developed countries, the prevalence of diabetes has been observed to rise in tandem with the rise in living standards and subsequent changes in lifestyle (Calvo-Hueros et al., 2021). DN affects about one-third of all diabetics (whether they have type 1 or type 2), causing major social and economic hardships (Akhtar et al., 2020). Kidney fibrosis, clinically defined as microalbuminuria resulting from an early rise in glomerular filtration rate (GFR) is the pathogenic basis of DN (Sharma et al., 2021). Various evidences have indicated that genetic susceptibility, dyslipidemia, hyperglycemia, and hypertension are all involved in the onset and DN progression. Even though DN is being recognized as a primary cause of morbidity and mortality in the majority of renal disorders, no therapeutic options directly target the pathophysiology of DN. On the contrary, elevated blood pressure and glucose levels are the primarily addressed issues. Nonetheless, fibrosis, oxidative stress, and inflammation have been continuously pointed out as the major links in the progression of DN, in the investigation that took place over the last 5 years, providing researchers with quite a few possible targets (Chen et al., 2021; Wu et al., 2021). Hence, the direction of anti-DN agent research has been shifted to focus on the multiple mechanisms related to DN. These include interruption of cell signal transduction pathways, targeting interrelated enzymes, and lessening in the extracellular matrix (ECM) aggregation (Cure and Cumhur Cure, 2020; García-Prieto et al., 2021). The drugs developed should have a high affinity for multifactorial pathogenesis, allowing them to limit ECM formation and hasten its disintegration. Although kidney diseases cause a significant burden upon the health care system, effective therapeutics for kidney disease are only a few, making kidney disease one of the most serious dangers in the twenty-first century (Zheng et al., 2020; Yan et al., 2021). As a result, novel techniques, drugs, and technology to identify and treat renal illnesses more efficiently, accurately, and conveniently are in high demand.

**FIGURE 1:**
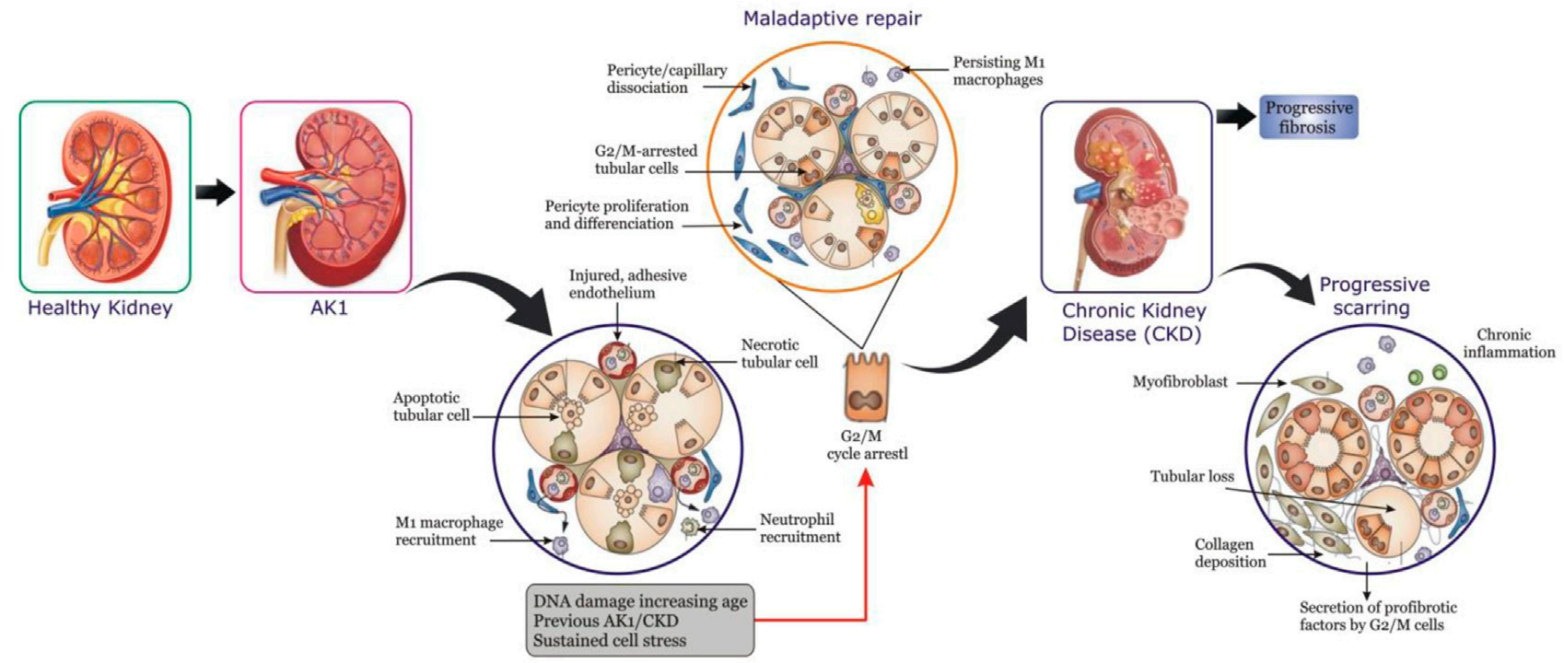
Maladaptive repair after acute kidney injury (AKI) can result in chronic kidney disease (CKD).

Nanomedicines and nanoparticles (NPs) have lately surfaced as effective diagnostic, imaging, and therapeutic agents for a variety of diseases and disorders (Afsharzadeh et al., 2018; Oroojalian et al., 2019; Oroojalian et al., 2020). A variety of kidney illnesses, including DN, chronic kidney disease (CKD; Figure 1), and acute kidney injury (AKI) can benefit from the use of NPs for diagnostic imaging and molecular targeting. Owing to their efficacy, specificity, and diversity nanomedicines and NPs may be able to address the challenges related to the treatment of renal diseases (Liu et al., 2019). However, despite the amazing promise of nano-based therapies for treating renal disorders, their use has been rather limited on account of the hurdles faced during systemic administration, and tailored distribution (Kamaly et al., 2016). These obstacles can be encountered in the bloodstream, as well as when entering the kidney and accessing the target areas in the real tissue. It is rather fortunate that recent outcomes (Zhou et al., 2014; Kamaly et al., 2016) have indicated ways for increasing NPs transport from the circulation to the kidneys, as well as to improve their retention in the kidney, potentially resolving the aforementioned issues. Nefzger et al. (1984) (Lien et al., 2021) first demonstrated that polymeric NPs made of poly (methylmethacrylate) (PMMA) are capable of reaching renal tissue *via* systemic circulation. In the 1990s, research on kidney-targeted drug delivery systems (DDSs) began, and in 1994, actinomycin D loaded poly (isobutylcyanoacrylate) NPs were developed as a targeted DDS for glomerular mesangial cells that could be concentrated in rat mesangium (Liu et al., 2019). The rapid expansion of nanomedicine, in general, has tremendously accelerated subsequent discoveries in this field of research. Targeted NPs have recently been established as prospective carriers for targeted therapeutic delivery in preclinical as well as clinical studies, in both academic, and industrial labs (Bidwell et al., 2017; Oroojalian et al., 2017). However, there are quite a few challenges that must be effectively handled before they can be widely evaluated in clinical studies.

The utilization of nano-technological methods for the treatment of renal disorders is the core subject of this review. Additionally, kidney-specific delivery strategies, as well as approaches for limiting NP accumulation in distant organs and ensuring optimal duration for blood clearance, have been taken into account. Besides, the impact of the NPs’ functional and physicochemical features on their capability to target specified kidney regions has been thoroughly examined.

## 2 Development of Diabetic Nephropathy

Aside from heredity, age, and obesity, at least two theories have been put forth for the occurrence and progression of DN: metabolic and hemodynamic problems, which include lipid and blood glucose metabolism disorders (Zhang et al., 2020). Furthermore, it is becoming increasingly obvious that fibrosis, inflammation, and oxidative stress are significant factors in the advancement of DN.

**FIGURE 2:**
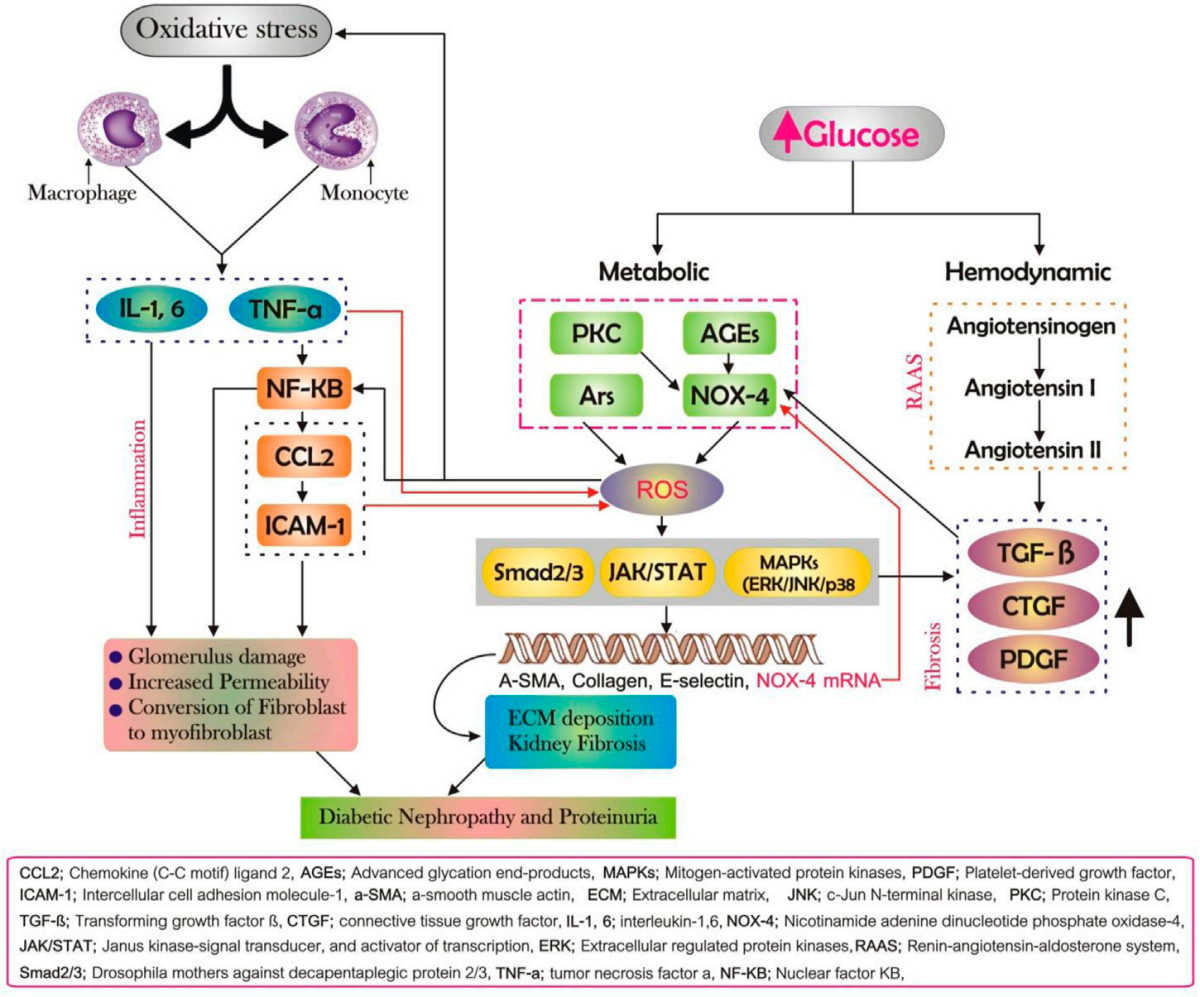
Diabetic nephropathy (DN)’s pathogenesis. Fibrosis, inflammation, and oxidative stress all have an involvement in the onset and progression of DN, and each of these pathways is intricately linked and reinforcing each other. A major part of DN is oxidative stress, which triggers inflammation and fibrosis, both of which increase oxidative stress; renal fibrosis occurs as a result of inflammation, exacerbating the inflammatory response.

Excessive formation of reactive oxygen species (ROS) is caused by stressors, for instance, high blood pressure and high blood glucose as diabetes progress. This disrupts the oxidation-reduction balance and damages the autoregulatory oxidation-reduction regulating mechanism, resulting in oxidative stress. Furthermore, multiple research studies have shown that ROS leads to direct and indirect damage to the renal interstitium as a consequence of oxidative stress in the case of long-term hyperglycemia conditions such as 1) renal vascular sclerosis; 2) increased vascular permeability; 3) structure and function damage; 4) activation of downstream mediators including nuclear factor-kB (NF-kB) and activator protein-1 (AP-1), p38 mitogen-activated protein kinases (p38 MAPK), extracellular regulated protein kinases (ERK). Subsequently, a set of cellular responses is triggered which are presumed to take part in the development of DN (as shown in Figure 2). The polyol pathway, increased production of advanced glycation end-products (AGEs), and activation of protein kinase C (PKC) and nicotinamide adenine dinucleotide phosphate oxidases (NOX) are the key ROS resources (Lv et al., 2015; Yabuuchi et al., 2020; Lien et al., 2021; Thallas-Bonke et al., 2021).

As previously stated, oxidative stress is the first stage of DN and initiates several pathogenic pathways. Fibrosis, on the other hand, is a fundamental and visible hallmark of DN, and inflammation seems to have a key function in the genesis and advancing of kidney fibrosis if left unchecked (Xu et al., 2020). The accumulation of macrophages, lymphocytes, mast cells, inflammatory cells (e.g., neutrophils), and dendritic cells within the renal tissue is intimately related to DN in animal models and live specimens with DN (Wen and Crowley, 2020). These inflammatory cells synthesize and secrete fibrogenic cytokines such as tumor necrosis factor-α (TNF-a), interleukin-1 (IL-1), and ROS in the local microenvironment, which directly harm the kidney architecture or stimulate the epithelial-to-mesenchymal transition (EMT) process, which tends to result in ECM accumulation (Qaseem et al., 2018). Other components engaged in the mechanisms of DN include chemokine C-C motif ligand 2 (CCL2), also referred to as monocyte chemotactic protein 1 (MCP-1). Renal fibrosis is a common result of kidney illnesses that results in severe loss of the normal structure and function of the kidney, regardless of whether the injury is caused by oxidative stress or inflammation. The thickening of the glomerular basement membrane (GBM), glomerular hypertrophy, and a rise in the mesangial matrix are all major pathogenic factors in kidney fibrosis and are the key features manifested by DN. Excess ECM is created as a result of prolonged cytokine stimulation, and its breakdown slows down. Moderate ECM is generated in normal circumstances to preserve normal cellular structure and function. Nevertheless, in case of a persistently existing injury, as an outcome of inflammation or oxidative stress, profibrotic cytokines are over-released to activate fibroblasts to express α-smooth-muscle actin (α-SMA), thereby contributing to ECM overproduction, and deposition in renal tissues. Transforming growth factor β (TGF-β) has a significant impact on downstream signaling, particularly the Smad pathway, during the fibroblast activation phase (Gewin, 2020). The pathogenic Smad signaling pathway is activated when TGF-β binds to receptors in mesangial cells, thereby upregulating the transcription of target genes including Mix2, speeding mesangial expansion, creating excessive ECM, and increasing kidney tissue fibrosis.

## 3 Traditional Therapies for Diabetic Nephropathy

When patients with microalbuminuria are diagnosed early on, treatment can be started to prevent disease development and lower the risk of end-stage renal disease (ESRD). The mainstay of DN treatment is the careful controlling of hypertension and hyperglycemia, as well as the use of medicines with specific renal benefits. Other potentially controllable risk factors should also be considered (Dunkler et al., 2015; Qaseem et al., 2018).

### 3.1 Glycemic Control

Dietary modification, weight loss, exercise, and drugs are all required to be an essential part of any treatment plan for hyperglycemia. Diabetic patients should make necessary lifestyle adjustments and use metformin as their first-line treatment. Drugs from a wide range of pharmacological classes were found to affect intermediate renal outcomes in larger studies. regardless of their glucose-lowering actions, may help decrease the progression to DKD (Marso et al., 2016a; Marso et al., 2016b; Groop et al., 2017). Dipeptidyl-peptidase-4 inhibitors and Glucagon-like peptide-1 receptor agonists, in particular, prevent albuminuria progression, while sodium-glucose cotransporter-2 inhibitors diminish renal disease progression and lessen the demand for kidney replacement therapy (Wanner et al., 2016; Groop et al., 2017; Heerspink et al., 2017). For individuals who are not able to attain their A1C level *via* lifestyle modifications and metformin, sodium-glucose cotransporter-2 inhibitors and glucagon-like peptide-1 agonists are advised as second-line therapy due to their inherent renal protection (Association, 2019a).

### 3.2 Blood Pressure Control

Systolic blood pressure should be lower than 140 mm Hg, and diastolic blood pressure should be under 90 mm Hg to ensure a lower risk of microvascular disease (including DN) (De Boer et al., 2017; Shibata et al., 2021). Lower blood pressure targets ranging from 130 to 80 mm Hg may be recommended for a few patients (e.g., patients with known DN or higher risk of atherosclerotic cardiovascular ailments) if they can be met without severe treatment side effects. The first line of treatment for hypertension in diabetic people should be lifestyle changes. This involves limiting salt intake (under 2,300 mg daily consumption), weight loss in the case of obesity, and staying active (Care, 2019). To reach target blood pressure, a patient-specific treatment strategy must be developed at the time of diagnosis and should be executed along with pharmacologic therapy (Bertsimas et al., 2020; Indriani et al., 2020).

The use of angiotensin receptor blockers (ARBs) and angiotensin-converting enzyme (ACE) inhibitors can help to slow down and stop the progression of DN (Zhu et al., 2021). ACE drugs, according to a 2012 Cochrane study, reduce the incidence of new-onset macroalbuminuria or microalbuminuria in diabetics with/without hypertension (Strippoli et al., 2005). In the same study, ACE inhibitors were found to minimize the risk of death in diabetic individuals when compared to a placebo. A large randomized controlled trial published in 2011 suggests Benicar^®^ (olemesartan) improves early microalbuminuria when compared to placebo to placebo (whereas both groups met their blood pressure goals). A follow-up study published in 2014 found that this benefit is maintained over time (Haller et al., 2011; Menne et al., 2014). Dual therapy with ARBs and ACE inhibitors provides a little additional benefit, although the threats of hypotension, hyperkalemia, and kidney failure increase (Makani et al., 2013). Moreover, aldosterone antagonists show better therapeutic benefits when used in conjunction with ARBs or ACE inhibitors, but they come with a high risk of hyperkalemia, therefore they must be recommended with caution. Similarly, thiazide diuretics and calcium channel blockers exhibit good cardioprotective effects but they do not appear to be as effective in preventing DN development (Bangalore et al., 2016).

### 3.3 Lipid Management

DN changes lipid metabolism, leading to a rise in the low-density lipoprotein cholesterol complexes and an enhanced risk of adverse outcomes associated with atherosclerosis. Although statin-based drugs do not affect the course of DN, it has been shown to lower cardiovascular events and death in individuals with non-dialysis dependent renal impairment (in the presence or absence of diabetes) (Tuttle et al., 2014). Since quite a few statins are processed by the kidneys, dosages should be lowered for patients whose eGFR is greatly reduced. In contrast, there is no need to change the doses of atorvastatin (Lipitor) prescribed. Statin usage in hemodialysis patients has yielded mixed outcomes, with reduced levels of relative benefits (Fellström et al., 2009). For diabetic patients of all ages and at all stages of DN, discussion and collaborative decision-making on the initiation as well as the continuation of statin therapy are recommended (Care, 2019).

### 3.4 Dietary Modification

Dietary changes have the potential to slow down the advancement of DN, although the evidence for specific therapies is conflicting. Evidence has shown that a protein-restricted diet (0.8 g per kg per day) can delay the loss of GFR and progression to ESRD in individuals with DN, therefore, the American Diabetes Association advises it (Association, 2019b). A Mediterranean diet, as well as the DASH (Dietary Approaches to Stop Hypertension) diet, can be advantageous. Fresh fruits and vegetables, whole-grain carbs, fiber, omega-3, and omega-9 fats, and fewer than 2,300 mg of salt per day are all part of these diets. Sugary, saturated fat-rich, and processed carbohydrate-rich foods should be avoided. Routine evaluation for phosphorus, potassium, and vitamin D changes in DN patients may lead to additional dietary changes.

## 4 Recent Nano-Technological Approaches for Targeting and Imaging of Diabetic Nephropathy

**FIGURE 3:**
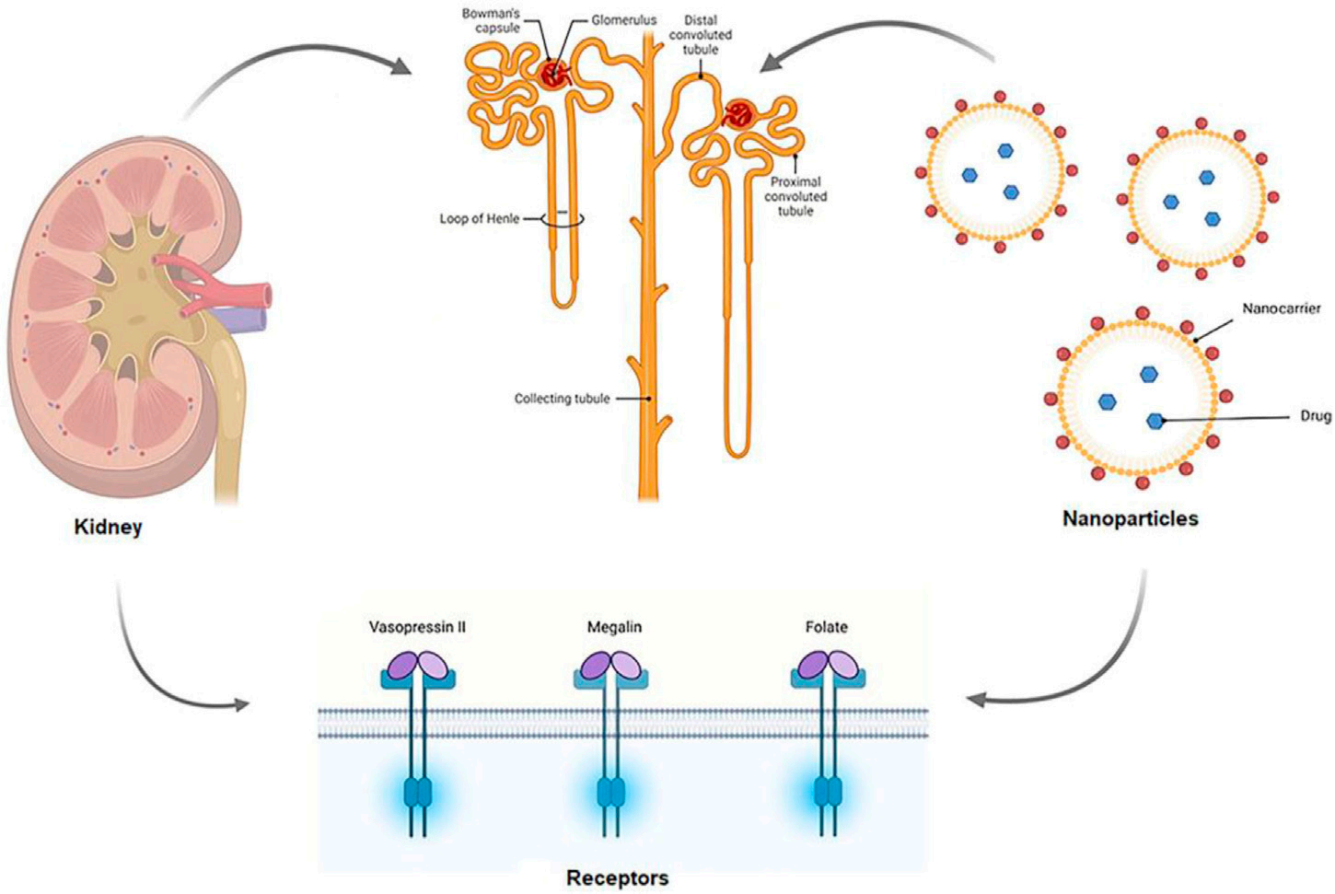
Schematic mechanism of kidney targeted drug delivery. The renal artery transports drug-carrying nanoparticles to the afferent arteriole, where they remain in the bloodstream or are filtered out of the blood by the kidneys in the glomerular capillaries for further processing. Renal components such as the endothelial cells, GBM, and glycocalyx can all be modified to assist in the selection of NPs for filtration. Following filtering, NPs can interact with podocytes in the Bowman’s lumen. The NPs are transported to the proximal tubule, where they interact with proximal epithelial cells and may be reabsorbed (Merlin and Li, 2021).

To treat nephropathic conditions, a high concentration of drugs must reach the kidney, which is naturally associated with a variety of adverse effects. Because of decreased tubular secretion and glomerular filtration processes in DN, administration of higher doses of the drugs does not ensure that the desired tissue receives the optimum amount of drug. Kidney-targeted drug delivery aids (shown in Figure 3) in the resolution of this issue (Hauser et al., 2021). In prodrug development, the use of macromolecular carriers or nanocarriers (e.g., liposomes) is commonly employed to deliver the drug to the intended site as shown in Table 1. Renal enzymes convert prodrugs to the active drug form. Assemblies of antibodies, proteins, and peptides are utilized as macromolecular carriers. The proximal tubular and glomerular mesangial cells are the primary targets when the drug is directed at the kidney.

**TABLE 1:** Nano-technological for targeting renal diseases and DN.

Nanoparticles	Size	Target/Location	Receptor/Antibody	% Selectivity/target specificity	References
Peptide-conjugated prodrug	—	Tubular and glomerular damage	Ang receptors	Selectively downregulated the AT_1_R level	Wyss et al. (2019)
HA-CUR prodrug	—	renal tubule epithelial cells	CD44 receptors	13.9-fold renal accumulation	Hu et al. (2018)
LMWC conjugated prednisolone	31 kDa	Renal	Megalin receptor	Significant accumulation in renal proximal tubules	(Yuan et al., 2009; Yuan et al., 2011)
LMW polycationic chitosan	450 nm	PTECs	—	>50% selectivity to PTECs	Gao et al. (2014)
PVD conjugated superoxide dismutase	73 kDa	Renal	—	Around 80% of polymer accumulated in the kidney	Yamamoto et al. (2004)
Carboxylated-PVP	—	Proximal tubular cells	—	Five-fold accumulation in proximal tubular cells	Kodaira et al. (2004)
PEP-PEA	100 nm	Proximal convoluted tubule	Ligand binding receptor	Specifically localized in renal *via* receptor-ligand complexes	Kim et al. (2012)
Protein/AuNPs-based sensors	51 nm	Podocytes	—	Selectively targeting the podocyte and regulating the level of nephrin and podocin	Bai et al. (2021)
ITSIONs	∼10 nm	renal medulla	RT1 anti-MHC Class II antibodies	RT1 ITSIONS indicates specificity for the renal medulla	Hultman et al. (2008)
Silica NPs modified with anti-CD11b	100 nm	Inflammed kidney	anti-CD11b	Enhanced anti-CD11b mediated uptake of silica NPs	Shirai et al. (2012)
Liposomal encapsulation of prednisolone	100–500 nm	Inflammed kidney	Glucocorticoid receptor	Specifically target the renal	Van Alem et al. (2018)
Mannose-PEG-DSPE	132 nm	Renal glomerular cells	Mannose receptor	Effectively target the renal glomerular cells	Yang et al. (2020)
PPE-AuNPs	20 nm	STZ-induced DN	—	PPE-AuNP suppressed STZ-induced renal toxicity	Manna et al. (2019)
Zinc oxide NPs	<50 nm	Renal tissues	—	≈2 fold renal accumulation	El-Khalik et al. (2021)
SeNPs	40 and 90 nm	Renal proximal tubular cells	—	SeNPs decreased the Bax level with increased Bcl-2 level in the kidney	Kumar et al. (2014)
Gold NPs	30 nm	Proximal renal tubular epithelial cells	—	AuNPs exhibited a nephroprotective role *via* the SIRT3-SOD2 signaling pathway	Yu et al. (2021)
Nanoconjugates of albumin	10 nm	Podocytes	Fc receptor (FcRn)	—	Wu et al. (2017)
Polymeric NPs	30–120 nm	Podocytes	—	Size-dependent internalization	Colombo et al. (2017)
Polycationic cyclodextrin NPs	70 nm	Glomerular mesangium	mannose receptor	Specifically target the Glomerular mesangium	Zuckerman et al. (2015)
Cyclodextrin-based siRNA NPs	60–100 nm	GBM	—	≈75% GBM accumulation	Zuckerman et al. (2012)
PLGA-PEG NPs	347 nm	Proximal tubules	—	26-fold renal selectivity	Williams et al. (2018)
Mesoscale NPs	400 nm	Renal proximal tubules	—	7-fold renal selectivity	Williams et al. (2015)
dDAVP-9r	204 nm	Inner medullary collecting duct	vasopressin V2 receptor	V2 receptor-mediated internalization in collecting duct	Jung et al. (2012)

### 4.1 Prodrug Nano-Carriers Approach

A prodrug is a drug that is unresponsive until it is activated by the action of an enzyme or by metabolism. Prodrugs modified with amino acids, folate, and sugar moieties have the potential to be used for renal targeting (Association, 2019b). There are specific receptors in the renal tubules, such as folate receptors and other enzymes, which are involved in the prodrug’s uptake. Wang et al. (1997) investigated the absorption of folate–diethylenetriamine pentaacetic acid conjugate. They noticed that the kidney has a high concentration of folic acid conjugates when compared to other organs. The impact of altering prednisolone on its absorption in HK-2 cell lines was observed by Li et al. (2019) (Lin et al., 2012). When compared to unmodified prednisolone, the glycated prednisolone demonstrated a greater than four-fold increase in sugar prodrug of prednisolone absorption. Using the tetrapeptide SS-31, Golshayan et al. developed a peptidase targeting peptide-conjugated prodrug (Wyss et al., 2019). Angiotensin receptors and aminopeptidase A are important regulators of renal function in the RAAS. The SS-31 tetrapeptide prodrug reduced AT1R mRNA expression while increasing AT2R expression. Prodrugs based on the SS-31 tetrapeptide act as aminopeptidase A substrates and avoid injury to the kidney. Curcumin and hyaluronic acid were linked in a hyaluronic acid–curcumin prodrug developed by Du et al. (Hu et al., 2018). Hyaluronic acid not only helped target the CD44 receptors (overexpressed on renal tubular cells in instances of injury), but it also enhanced the solubility of the curcumin. Biodistribution studies revealed that the hyaluronic acid-curcumin prodrug accumulates disproportionately in the kidney to the other organs.

### 4.2 Macromolecular Based Nano-Carriers

Macromolecular carriers have been reported to successfully carry the drugs to the kidneys. Biologically active low-molecular-weight proteins (LMWP) (less than 30 kDa) are commonly employed. Insulin (peptide hormones), lysozymes (enzymes), and light chain immunoglobulin (immune proteins) are all examples of LMWPs. These are reabsorbed in the renal tubules after being filtered at the glomerulus. The presence of LMWP overrides the drug’s kinetics (Desai et al., 2021). Peptide, disulfide, ester (poly-hydroxy acids), and amide (oligopeptides and lysine-amino group) bonds can be used to combine LMWP with drugs to produce the conjugate, which can then be broken down to release the drug *via* enzymatic or chemical hydrolysis. Because LMWPs contain several active groups that aid in LMWP self-aggregation, careful reaction control is required during the synthesis of drug conjugate with LMWP (Ramos et al., 2020).

#### 4.2.1 Polymeric Nano-Carriers

In recent times, the significance of polymers in drug delivery has become high because of their biocompatibility, which allows for precise and controlled delivery. Polymer distribution, functional groups, charge, molecular weight, and accumulation throughout the body are all important factors that affect their therapeutic efficacy and targeting. A few polymers have been identified as drugs transporters at the renal site. With increasing molecular weight, polymer clearance generally declines. The drug-polymer complex has several advantages, including improved specificity, fewer side effects, and improved efficacy (Pradeepkumar et al., 2018). Low molecular-weight chitosan (LMWC) is frequently used to develop DDSs for targeting renal-related diseases. When compared to lysozyme-drug conjugates, LMWC-drug conjugates have been found to have a faster renal clearance and thus a safer pharmacokinetic profile (Zhou et al., 2014; Sarko and Georges, 2016).Yuan et al. (2009) and Yuan et al. (2011) designed a study to determine the chitosan’s renal targeting capability by conjugating prednisolone to LMWC. Prednisolone was delivered to the kidneys more efficiently owing to this conjugate’s specific accumulation in the kidney. Gao et al. (2014) used an LMW polycationic chitosan and formed nanoparticles with polyanionic siRNA duplexes of approximately 450 nm in size. Using an injection of the LMW chitosan nanoparticles loaded with siRNA duplexes specific for water channel aquaporin 1 (AQP1) into mice, they were able to target more than 50% of the proximal tubule epithelial cells (PTECs). Megalin was used to target PTECs, and the expression of AQP1 was reduced in the targeted PTECs. This proof of principle experiment demonstrated the potential of LMW chitosan siRNA duplex nanoparticles.

A conjugate of superoxide dismutase with poly (vinylpyrrolidone-co-dimethyl maleic acid) (PVD) can be used as a drug molecule in renal diseases (Yamamoto et al., 2004). PVD has a high affinity for the kidneys and accumulates in the kidneys. When combined with superoxide dismutase, PVD has the potential to build up in the kidneys and be used to treat kidney disease. As suggested by an *in vivo* investigation based on male BALB/c mice, the distribution of the polymer is influenced by the molecular weight and charge of the PVD. *In vivo* results showed that 6–8 kDa molecular weight polymers accumulate rapidly in the kidneys, with around 80% of the polymer being selectively delivered into renal tissues following intravenous delivery. PVD’s negative charge indicates how long it takes for it to be eliminated from the kidneys. The renal targeting capability of different polyvinylpyrrolidone (PVP) derivatives was investigated by Tsutsumi et al. (Kodaira et al., 2004). They observed that carboxylated-PVP was more efficiently immersed by proximal tubular cells in comparison to sulfonated-PVP *in vivo*. The results of this investigation show that carboxylated-PVP possesses excellent renal targeting characteristics.

#### 4.2.2 Peptides and Antibody Ligands Based Nano-Carriers

Peptides are amino acid polymers that are easy to be synthesized and can be easily degraded in the body. Targeted delivery of drugs can be accomplished by the use of specific interactions of receptors with peptides. Phage display technology advancements have raised the number of peptides available for targeted delivery to the kidneys. Polyethyleneimine was previously thought to have a strong transfection capacity but also significant cytotoxicity. To circumvent the polyethyleneimine’s cytotoxicity, the use of poly (ester amine) (PEA) is preferred because of its low cytotoxicity and high transfection characteristics. Cho et al. designed a kidney-targeting system by coupling LTCQVGRVH, a kidney-targeting peptide, and with PEA. They discovered that PEA-peptide conjugation displayed selective transport of the HGF gene toward hydronephrosis in rats with unilateral ureteral obstruction (Kim et al., 2012). This PEA-peptide combination has decreased cytotoxicity and could be employed as a nonviral gene delivery vehicle. SS-31 is a tetrapeptide that targets mitochondria and can forage ROS. Hou et al. (2016) studied the effects and molecular mechanism of SS-31 peptide on mouse mesangial cells subjected to a concentrated glucose environment and a DN model. In diabetic mice kidneys, SS-31 reduced Bax expression and apoptosis in renal cells while reversing Bcl-2 expression. SS-31 also downregulated the thioredoxin-interacting protein (TXNIP), Nox4, and TGF-1 expression, whereas it activated the NADPH oxidase, CREB, and p38 MAPK expression in DN. Wang et al. (2019) synthesized rhein-loaded lipo-nanoparticles composed of a polycaprolactone-polyethyleneimine core wrapped with a lipid layer carrying a kidney targeting peptide. These lipo-nanoparticles demonstrated significant targeting toward the kidneys and prolonged retention in the kidneys significantly enhanced the rhein effect in DN and declined the DN progression. Production of protein-protein/gold nanoparticle-based sensors has been demonstrated by Bai et al. (2021). to monitor the progression of renal disease in murine experimental Adriamycin Nephrosis, a model for human Focal Segmental Glomerulosclerosis, the main cause of adult and pediatric nephrotic syndrome. Adriamycin induces a podocyte injury, leading to proteinuria and, subsequently, glomerulosclerosis. Bai constructed a sensor by introducing amino head groups to gold nanoparticles (AuNPs) through fluorophore proteins for monitoring and evaluating the process of Adriamycin Nephrosis. Using the linear discriminant and principal component analyses, the protein/AuNPs-based sensors classified the nephropathy progression into seven stages and assessed the Huangkui capsule’s protecting potential. A significant difference in podocin and nephrin expression as well as the morphological variations in podocytes further validate the reliability of the sensing method. This sensing approach may be used to track nephropathy progression and assess protective medications, thus speeding up research into clinical diagnosis and nephropathy treatment.

Active targeting is a very efficient method of targeted delivery that makes use of a homing moiety that is a component of an adaptive system such as antibodies. Antibodies are large protein-based molecules that are produced by the immune system in response to antigens. Antibodies with greater than 150 kDa molecular weight are inefficient as proximal tubular cell targeting carriers because they cannot cross the glomerular filtration. Therefore, antibody fragments with less than 50 kDa molecular weight appear to be a potential targeting ligand for kidney cells (Chen et al., 2020). Hultman et al. (2008) successfully developed and applied immunotargeted superparamagnetic iron oxide nanoparticles (ITSIONs) for magnetic resonance diagnostics and possible drug administration for nephropathy *in vivo*. ITSIONs are biocompatible, stable, targeted nanoparticles composed of an iron oxide core and wrapped with functionalized phospholipid carrying an antibody to a specific target antigen. Antibodies against MHC Class II RT1 were utilized to specifically focus on the rat renal medulla, a region of the kidney where MHC Class II, an inflammatory protein, is highly expressed. An increase in the binding of the NPs to the renal medulla implies selectivity for the medulla, which could lead to a proper diagnosis or the delivery of therapeutics to this region. Yasuhiko et al. modified silica NPs with anti-CD11b for selective diagnostics of renal diseases (Shirai et al., 2012). Renal inflammatory imaging was performed after the intravenous injection of modified silica NPs into mice with unilateral ureteral obstruction (UUO). The accumulation of modified silica NPs was proportionately higher in the UUO kidneys than in the non-inflamed kidneys or normal kidneys. The authors conclude that silica NPs modified with anti-CD11b are promising nanoprobes for high-intensity imaging of inflammatory sites.

### 4.3 Liposomes

Liposomes are composed of one or more phospholipid bilayers. They have a diameter ranging from 20 to 10,000 nm and can be produced artificially by utilizing a variety of techniques (Alavi et al., 2017). Macrophages can actively pick up the particles, or they can be passively absorbed into a cell’s membrane. Liposome vesicles are used to transport therapeutic molecules and can be loaded with bioactive compounds. The vesicle half-life and distribution *in vivo* can be controlled by modifying the vesicles. Small capillaries can be excluded from liposome distribution by using bigger-sized vesicles, which can confine the liposomes’ dispersion to larger blood capillaries. If polyethylene-glycol is coated onto the liposome’s surface, the vesicle’s half-life can be lengthened by escaping from the macrophages and more drugs can be released over time. To promote contact with certain cell types or receptors, liposomes can be modified with peptides containing antibody fragments or binding domains. Liposomes can transfer nucleotides as well as medicines or small compounds, making them a useful tool in gene therapy, and gene editing strategies.

In gene therapy approaches, liposomes have been employed as carriers to treat experimental congenital defects, for example, acute kidney injuries, polycystic kidney disease, or Alport’s syndrome (Rubin and Barry, 2020). Van Alem et al. (2018), have demonstrated that liposomal encapsulation of prednisolone can be used for the targeted delivery of glucocorticoids to the kidney. Using an experimental model for renal ischemia-reperfusion injury, they were able to show that liposome-encapsulated prednisolone accumulated in the inflamed kidney and increased the presence of anti-inflammatory macrophages, suggesting a reduction in proinflammatory macrophages. A Mannose-PEG-DSPE (Polyethylene glycol-1, 2-Distearoyl-sn-glycero-3-phosphoethanolamine)-modified liposome was developed as a vehicle for the targeted delivery to renal glomerular cells *via* the Glucose Transporter (GLUT) (Yang et al., 2020). In diabetic nephropathy, GLUT is highly expressed in glomerular mesangial cells and renal vascular smooth muscle cells. The study by Yang and colleagues demonstrated that mannose-PEG-DSPE modified liposomes can be used as a long-term vehicle for targeted delivery in a rat model of diabetic nephropathy.

Liposomes’ poor affinity for the kidney is also employed to reduce the nephrotoxicity of some drugs. The liposome-encapsulated antifungal drug Amphotericin B (liposomal AmB), for example, has been shown to have lower nephrotoxicity (Faustino and Pinheiro, 2020). For the antibiotic vancomycin, the cytostatic agent cisplatin, and other platin-based anticancer medicines, similar techniques have been devised (Hauser et al., 2021).

### 4.4 Metal or Inorganic Nanoparticles

When using NPs to target the kidneys, the particle size plays a significant role in determining the target region in the kidney. Particles small enough (5–7 nm) to pass through the glomerular filtration barrier can be targeted and reabsorbed in the tubular region. Particles having dimensions of 30–150 nm cannot pass through the glomerular filtration and persist in blood circulation (Kamaly et al., 2016). The particle size of 80 nm resulted in the greatest glomerular accumulation. There are, however, exceptions. For instance, the liver can eliminate ultra-small particles (up to 2 nm) because they cannot pass through the glycocalyx. Mesoscale NPs (MNPs) (up to 400 nm) were observed in proximal tubular cells, apparently delivered there through endocytosis as their size is larger than fenestrations. Manna et al. (2019) synthesized and investigated the effect of pomegranate peel extract-stabilized gold nanoparticles (PPE-AuNPs) on the mice with the streptozotocin-induced DN model. In hyperglycemic animals, PPE-AuNPs significantly decreased serum TG and TC, renal toxicity indices, and fasting blood glucose. PPE-AuNPs normalized streptozotocin-induced pancreatic-cell dysfunction, as evidenced by an increase in plasma insulin levels. The PPE-AuNPs treatment dropped the production of iROS and LPO, restored the streptozotocin mediated inhibition of endogenous antioxidant response. Furthermore, the application of PPE-AuNPs reduced the hyperglycemia-mediated augmentation of protein glycation, followed by NOX4/p-47phox activation. Histological and immunohistochemical analysis revealed that PPE-AuNPs were protective against streptozotocin-induced renal fibrosis and glomerular sclerosis. Additionally, it decreased the proinflammatory burden by modulating the MAPK/NF-_k_B/STAT3/cytokine axis. Simultaneously, activation of Nrf2 *via* PI3K/AKT was observed following PPE-AuNPs application, enhancing the antioxidants responses, and maintaining hyperglycemic homeostasis. El-Khalik et al. (2021) studied the mechanistic reno-protective effects of zinc oxide NPs in streptozotocin-induced DN. After 6 weeks of treatment, zinc oxide NPs significantly improved histopathological, biochemical, and renal parameters. Additionally, zinc oxide NPs significantly decreased TXNIP gene expression and increased Nrf2-DNA-binding potential, resulting in redox status restoration. Zinc oxide NPs increased autophagy activity, decreased AGE levels, and inhibited inflammasome activation through IL-1β reduction and NLRP3 downregulation. They concluded that zinc oxide NPs may be a promising agent for decelerating the progression of DN through the Nrf2/TXNIP/NLRP3 inflammasome signaling pathway. Kumar et al. (2014) investigated the protective effect of SeNPs on diabetic nephropathy progression. SeNPs exerted biological activity in streptozotocin-induced diabetic nephropathy by decreasing oxidative stress, increasing the activity of the longevity protein Sirt1, the cytoprotective protein Hsp-70, and modulating the expression of the anti-apoptotic protein Bcl-2 and the apoptotic protein Bax in the apoptotic kidney.

Yu et al. (2021) synthesized gold NPs by a hydrothermal method to evaluate their anti-DN effects by mitigation of oxidative stress. They investigated the effects of 50 mM high glucose treatment on the toxicity of HK-2 cell lines as well as the protective effects of hydrothermally prepared gold NPs on AGEs and free radical formation. Gold NPs with a size of about 30 nm and that are colloidally stable at pH 7.4 were found to significantly reduce high glucose-induced toxicity in HK-2 cell lines by enhancing cell viability and decreasing the formation of AGEs and free radicals. Gold NPs also downregulated the expression of Caspase-3 and Bax/Bcl-2 at both the protein and mRNA levels. Hence the study indicated that the gold NPs can be considered as an effective approach towards the prevention of DN progression.

Lu et al. (2012) developed a nanoparticle transfection method for the targetted delivery of siRNA to treat systemic hypertension, which originates in the renal tubules and vasculature, as well as for the treatment of atherosclerotic lesions. They designed a galactose polyethylenimine glycoprotein urethane (GPE) that binds anti-angiotensinogen (AGT) siRNA and demonstrated that 72 h following the transfection of GPE-AGT siRNA nanoparticles into spontaneously hypertensive rats, AGT protein expression in the liver was considerably reduced. AGT and its cleavage product, angiotensin II, were found to be lower in the blood. When compared to the baseline, blood pressure dropped considerably. They also discovered that atherosclerotic plaques had lessened and renal function had remained steady. GPE nanoparticles can be used to control the renin-angiotensin system *via* particular siRNA, as suggested by the study.

## 5 Renal Glomerulus as a Targeting Site

Kidney-targeted therapy may increase the efficacy and safety of therapeutic drugs. The glomerulus and tubule system comprises the nephron, the kidney’s structural, and functional unit. The glomerulus is composed of a cluster of blood capillaries and the mesangium. The glomerular filtration barrier is composed of three layers: glomerular endothelial cells (GECs), GBM, and podocytes. The GECs form a dense network of fenestrations and transcellular holes ranging from 60 to 80 nm filled with endothelial glycocalyx (Satchell and Braet, 2009). They prevent circulating plasma constituents from trying to enter endothelial cell membranes *via* a negative charge and a filamentous network (Curry and Adamson, 2012). The GBM is a 300 nm wide, continuous membrane with pores that are 2–8 nm wide, which is composed of heparan sulphate proteoglycans (negatively charged), nidogen, laminin, and collagen IV (Kanwar and Farquhar, 1979; Ogawa et al., 1999). These layers form an interconnected meshwork that filters small molecules according to their size and charge. The podocytes are tightly attached to the GBM and have interdigitating foot processes, that form filtration slits of 20–30 nm in width (Lahdenkari et al., 2004). Hence, the various approaches for targeting nephrons are podocytes, mesangial cells, GBM, and proximal tubule (Zuckerman and Davis, 2013).

### 5.1 Podocyte-Targeted Nano-Carriers

Podocytes, also known as the kidney’s glomerular epithelial cells, are differentiated cells of the kidneys that have complex cell structures involving enormous foot processes (FPs) and mutual connection. By draping the GBM, the FPs form highly selective filtering machinery after separating from the cells (Greka and Mundel, 2012). Podocytes make use of these filtration incisions with a size cutoff of around 10 nm to stop plasma proteins from entering the urine filtrate, enabling only tiny solutes to pass through. Podocyte function must be ensured properly to ensure optimal renal function and glomerular filtration (Mathieson, 2012). DN initiates albumin leakage because of a decline in podocytes, self-effacement of podocyte FPs, and failure of filtration incision (podocin and nephrin). Damage to podocytes and the resulting albuminuria have been seen as essential events in DN (Lin and Susztak, 2016). Many studies have been conducted to find novel targeted therapeutics that can effectively treat DN by preventing or reversing the consequences of podocyte injury.

Wu et al. (2017) reported the development of nanoconjugates by linking albumin (as a carrier) to methylprednisolone *via* an amide bond (an acid-sensitive bond) to avoid the side effects associated with glucocorticoid therapy in patients with nephrotic syndrome (a result of DN). The nanoconjugates had monodispersed and homogeneous size distribution, with a size of about 10 nm, could pass through the glomerular filtration barrier. Albumin is a portion of the neonatal Fc receptor (FcRn) over-expressed in podocytes, allowing for localized administration into cultured human podocytes. The nanoconjugates displayed dose-dependent and receptor-mediated absorption in human podocytes. Cellular lysosomes cleave the acid-sensitive connection, facilitating the release of drugs from the nanoconjugates after cellular absorption. Colombo et al. (2017) synthesized a series of biocompatible polymeric NPs with specific surface properties and controllable sizes with the same goal of delivering glucocorticoids locally to treat injured podocytes. Copolymerization with polymerizable methacryloyl surfactants produced negatively charged, positively charged, and neutral poly (-caprolactone) based NPs with particle diameters ranging from 30 to 120 nm. The size and surface charge of the nanoparticles affected their cytotoxicity and absorption. The persistent therapeutic efficacy of the entrapped drug was exhibited on podocyte cultures, as dexamethasone effectively repairs damaged podocytes.

### 5.2 Mesangial Cells-Targeted Nano-Carriers

Mesangial cells are flattened cylindrical-shaped cells that make up the glomerulus’ central stalk. They work along with podocytes and endothelial cells to form a functioning glomerular unit. The contractile characteristics of mesangial cells modulate the surface of glomerular filtration and the glomerular capillary flow (Scindia et al., 2010). Any damage to mesangial cells, whether direct or indirect, is a key function in the growth of ESRD, resulting in hypertrophy, mesangial enlargement, extracellular matrix buildup, and cell proliferation (Tung et al., 2018). The potential of selectively regulating mesangial cells with a therapeutic drug administered externally is appealing as a novel treatment approach because they are important in the onset and DN progression (Alpers and Hudkins, 2018).

Zuckerman et al. (2015) developed siRNA-loaded NPs from polycationic cyclodextrin for targeted delivery of siRNA to the glomerular mesangium. Since mannose receptor (MR) is only expressed in the mesangium, siRNA-loaded NPs were able to remain in the glomerulus for longer when mannose was used as the surface targeting ligand. The significant uptake of targeted siRNA-loaded NPs by mesangial cells was qualitatively confirmed through epifluorescence experiments. After the intravenous injection of siRNA-loaded NPs, gene suppression in the mesangium was assessed by measuring the level of production of downregulated green fluorescent proteins in mesangial cell lines and transgenic mice with elevated EGFP-expression. This study exhibited that both human and mouse mesangial cells rapidly internalized siRNA-loaded NPs instead of free siRNA. Surface targeting ligands can help mesangial cells by allowing better absorption of siRNA-loaded NPs. The NPs accumulate within the renal corpuscle in a size-dependent manner due to their particular anatomy. NPs with a size of 80–150 nm passively aggregate in the fenestrated endothelia pore during their circulation. This size-dependent accumulation opens up the possibility of developing new therapeutic approaches.

### 5.3 Glomerular Basement Membrane-Targeted Nano-Carriers

**FIGURE 4:**
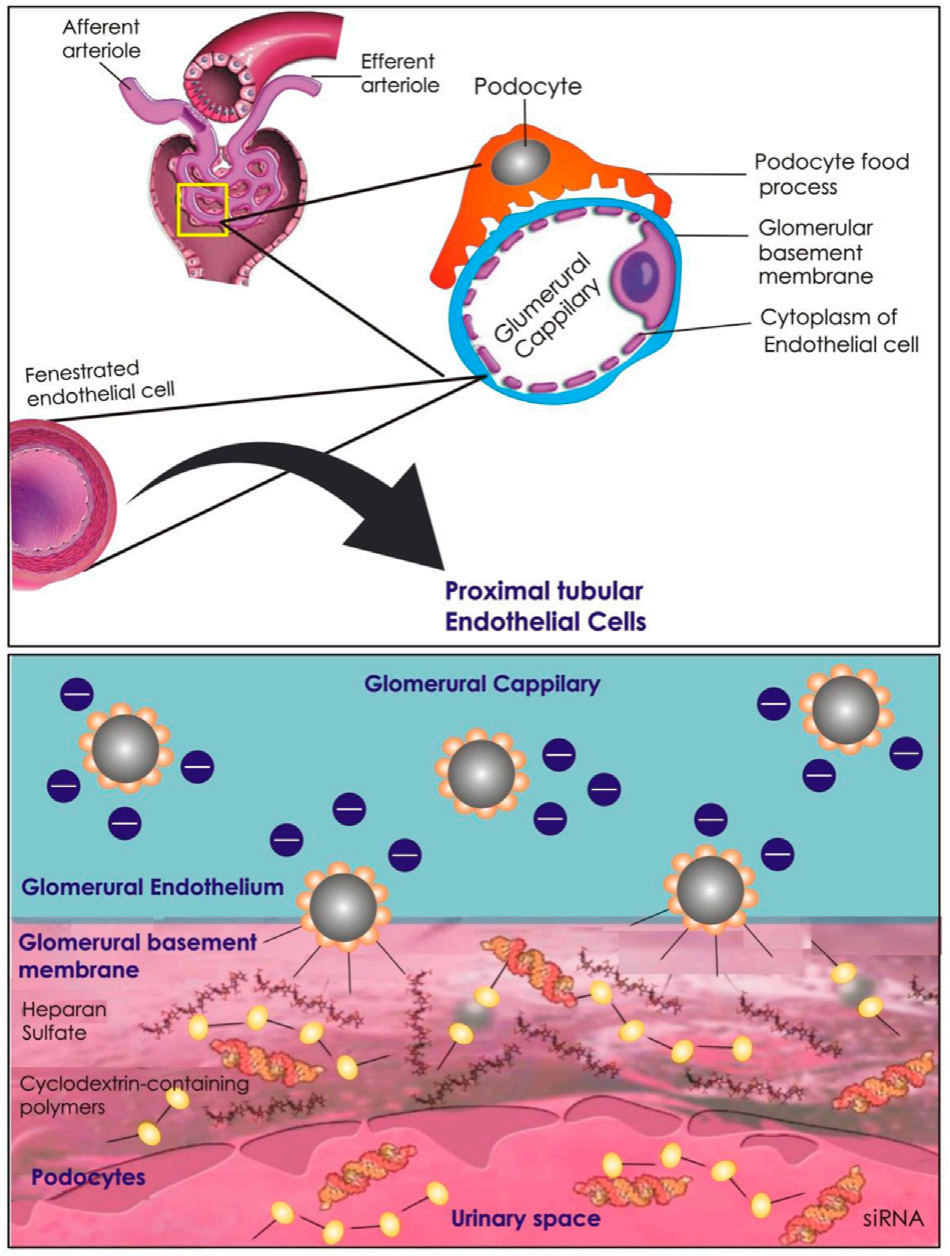
Schematic illustration of siRNA-loaded NPs accumulation and dissociation in the GBM. NPs pass through the fenestrations in the glomerular endothelial cell lining and enter into the GBM. At the GBM, the NPs are disassembled due to the high amounts of haparan sulfate. The NPs may then pass through the GMB, and after podocyte filtration, reach the Bowman’s capsule.

A three-layered extracellular matrix GBM (Figure 4) is formed when the membranes of glomerular endothelial and podocytes come together (Li et al., 2019). GBM’s composition is complicated by the inclusion of approximately 140 different proteins (verified by proteomic study of isolated human GBM). TINAGL1, heparan sulphate proteoglycans (perlecan and agrin), nidogen-1, laminins (γ1, β2, and α5), and collagens (types I, IV, VI, and XVII subunits) are the most commonly occurring proteins among them (Miner, 2012). Collagen IV is the primary structural protein, forming a cross-linked network with the fibrous lattices and heterogeneous holes that provide support in the linking of further structural components. The GBM is a critical element of the glomerular filtration barrier, acting as a transitional sieve and an associated structure that permits the podocytes and glomerular endothelium to function appropriately (Miner, 2011).

Any structural damage to the GBM might cause anomalies and a lack of size selectivity, which can lead to proteinuria and albumin leakage. In the diabetic environment, the podocyte-GBM interaction is disrupted, with GBM thickness serving as an early indicator of DN development. Positively charged NPs concentrate rapidly in the GBM, a factor aiding drug delivery. The anionic character of the basement membrane, which originates from the existence of a considerable quantity of heparan sulfate carrying glycocalyx, is one probable explanation (Scott and Quaggin, 2016). Zuckerman et al. (2012) reported positively charged cyclodextrin-based siRNA NPs (potential, +10 mV) with specific anionic GBM interactions. TEM analysis showed that after siRNA NPs delivery, the kidney tissue had an abundance of dark-staining, round globules that were consistent with the size and form of siRNA NPs. Most NPs were located within the lamina rara interna, while a few of the smaller NPs were located within the limina rara externa. The accumulation of the siRNA NPs was also observed along the glomerular capillary walls by the corresponding high-intensity siRNA fluorescent signals in confocal microscopy studies. These findings established that intact NPs in circulation crossed the glomerular endothelial fenestrations and accumulated in the GBM. In earlier work, Bennett et al. (2008) made use of the electrostatic attraction between anionic GBM and cationic NPs to deliver metal oxide NPs as a contrast agent for identifying the breakdown of the basement membrane during illness. *Ex vivo* and *in vivo* MRI revealed selective localization of cationic ferritin but not native ferritin. Electron microscopy and immunofluorescence revealed that this cationic ferritin accumulation occurred exclusively within the GBM. MRI revealed a decreased accumulation of cationic ferritin in single glomeruli during focal and segmental glomerulosclerosis, but a dispersed accumulation of cationic ferritin in the kidney tubules was observed because of the cationic ferritin leaking from the glomerulus. Cationic contrast agents can be used to target the basement membrane and look for changes in the basement membrane in disease.

### 5.4 Proximal Tubule-Targeted Nano-Carriers

Reabsorption of the glomerular filtrate, which includes water, essential ions, and small molecules, is carried out by the proximal tubule of the nephron. Almost all the small peptides, amino acids, and glucose that have been filtered are reabsorbed in nephrons. The renal proximal tubular epithelial cell (PTEC) portrays a critical role in the pathophysiology of DN (Tang and Lai, 2012). PTECs coordinate kidney injuries in the course of DN by triggering major fibrogenic and proinflammatory signaling pathways through the activation of numerous important DM substrates. Fibrosis in renal tubules is caused by the accumulation of fibronectin, AGEs, and other inflammatory mediators. Tubulointerstitial inflammation can be triggered by microalbuminuria activating the PTECs (Gewin, 2018). Advanced tubulointerstitial fibrosis is a widely known cause of chronic renal failure in all kidney disorders.

Gao et al. (2014) developed siRNA-loaded chitosan NPs as a conceptual design to target PTECs for suppressing genes overexpressed in PTECs during DN. While siRNA therapy has garnered considerable attention for its specificity and efficacy, its therapeutic utility remains challenging due to unsatisfactory circulation time and the dependence of its biodistribution primarily based on its carriers. By exploiting PTECs’ affinity for LMW chitosan through receptor megalin, a specific distribution of chitosan polyplex NPs in PTECs has been confirmed by immunohistological analysis and fluorescence bioimaging. Furthermore, the NPs successfully suppressed AQP1 in PTECs via absorption through the endocytosis of a megalin-dependent pathway. Williams et al. (2018) reported mesoscale NPs composed of a diblock copolymer (PLGA-PEG) for targeting the kidneys. The NPs were found to have a 26-fold higher efficacy in the kidneys (particularly in the proximal tubules) than in other organs. The NPs were shown to be biocompatible and safe (as expected by the incorporation of FDA-approved polymers that constituted the NPs). In another study, Williams et al. (2015) reported cationic and anionic mesoscale NPs composed of PLGA functionalized with PEG. For biodistribution studies, the NPs were loaded with a fluorescent dye. After the injection of cationic or anionic mesoscale NPs into mice, images were obtained daily for the first 7 days, then biweekly for the next 3 months. Fluorescence *in vivo* biodistribution was used to track the degradation and localization of NPs over time. NPs were found to be concentrated in both the kidneys and the chest region. The analysis confirmed that the fluorescence intensity in the kidneys was significantly higher than in other organs. Fluorescence and computed tomography imaging of the kidneys of a mouse treated with anionic mesoscale NPs confirmed their localization and relatively uniform distribution throughout the kidneys.

### 5.5 Renal Collecting Ducts Targeted Nano-Carriers

High glucose concentrations cause-specific cellular effects in the kidney, affecting many different types of cells, including tubular and collecting duct systems (Vallon and Komers, 2011). Vasopressin levels rise in diabetes and have been linked to the development of DN *via* V2 receptor (V2R) activation in a type 1 diabetes experimental model (El Boustany et al., 2017). It has been suggested that the vasopressin II (V2) receptor, a G protein-coupled receptor (GPCR) with seven transmembrane domains, would be beneficial for targeted drug administration *via* active targeting in the epithelial cells lining the connecting tubule, distal tubule, and collecting ducts (Robben et al., 2004), Megalin receptor-mediated endosomal absorption of various drugs and safe release of the active substance from endosomes are important in renal fibrosis (Nastase et al., 2018). While characterized NPs may be able to accumulate passively in the kidneys, active targeting NPs such as peptides and antibodies are also being studied to improve renal targeting (Nastase et al., 2018). In a mouse model of type 2 diabetes, it was recently demonstrated that vasopressin contributes to albuminuria and glomerular hyperfiltration *via* the V2R. It establishes the causal relationship between vasopressin and renal damage in diabetic patients (El Boustany et al., 2017). To tackle diabetes and Polycystic Kidney Disease, a highly promising technique would be to guide therapeutic payloads to cells overexpressing V2R. Jung et al. (2012) utilized the peptide vasopressin analogue desmopressin (dDAVP) conjugated with nine D-arginines (dDAVP-9r), YFQNCPdRGGGGGRRRRRRRRR, serving as a peptide delivery platform tp deliver siRNA against aquaporins to collecting duct cells (Jung et al., 2012). FITC-siRNA/dDAVP-9r was found to bind to Madin-Darby Canine Kidney (MDCK) and pig epithelial-like kidney cells (LLC-PK1) *in vitro* at a level one order of magnitude higher than when FITC-siRNA alone or FITC-siRNA/9r was used. In monkey kidney (Cos-7) cells, which are negative for V2 receptors, no significant differences in cellular uptake of FITC-siRNA/9r and FITC-siRNA/dDAVP-9r were found, indicating that targeting the V2 receptor was required for cellular uptake of the payload siRNA (Rothbard et al., 2002).

## 6 Conclusion and Future Perspective

Type 1 and 2 diabetes mellitus is becoming a global impediment because of the prevalence of DN. It is essential to understand the molecular pathways and targets for targeted delivery in DN because DN is a complex condition that involves multiple mechanisms. Nano-formulations are a modern concept for efficiently delivering drugs to their intended sites. Numerous nano-formulations have been investigated for targeting delivery at different sites. Different carriers bearing various ligands have also been developed for efficient and targeted delivery of therapeutic or diagnostic agents to the kidney’s tissues.

Formulation scientists face a difficult task in designing an ideal platform for therapeutic drug delivery to the kidney’s tissues for DN treatment, given the kidney’s propensity to flush pharmaceuticals out of the body. As evidenced by the abundance of NPs already in the market and more are being tested in clinical trials to see if they can be approved, the field of nanomedicines will almost certainly hold a large market share soon. In the case of DN, nanomedicine-based techniques with designed biophysical features allow for enhanced interaction with kidney cells and improved cellular uptake inside proximal tubule cells, mesangial cells, podocytes, GBM, and epithelial cells resulting in improved retention. Valuable pioneering investigations have already begun, but additional targeted pharmacokinetic and pharmacodynamic research in clinical states that mirror typical albuminuria, as well as the modified GFR found in DN, are needed to assess nanomedicine’s relevance. Understanding the structural and functional aspects of the kidney and insights into the pathophysiology of DN can be used to improve drug delivery methods for clinical therapy and the study of DN. It’s high time for nano-technological researchers and nephrology professionals to work together to devise promising nanomedicine-based methods for the targeted therapy of DN.

## Glossary

GPE: galactose polyethylenimine glycoprotein urethane: galactose polyethylenimine glycoprotein urethane
AGT: anti-angiotensinogen
DN: Diabetic nephropathy
GFR: glomerular filtration rate: extracellular matrix
GFR: glomerular filtration rate: extracellular matrix
NPs: nanoparticles
AKI: acute kidney injury
CKD: chronic kidney disease
PMMA: poly (methylmethacrylate)
DDSs: drug delivery systems
ROS: reactive oxygen species
NF-kB: nuclear factor-kB
AP-1: activator protein-1
p38 MAPK: p38 mitogen-activated protein kinases
ERK: extracellular regulated protein kinases
AGEs: advanced glycation end-products
PKC: protein kinase C
NOX: nicotinamide adenine dinucleotide phosphate oxidases
TNF-a: tumor necrosis factor-α
IL-1: interleukin-1
EMT: epithelial-to-mesenchymal transition
CCL2: chemokine C-C motif ligand 2
MCP-1: monocyte chemotactic protein 1
GBM: glomerular basement membrane
α-SMA: α-smooth-muscle actin
TGF-β: Transforming growth factor β
ESRD: end-stage renal disease
ARBs: angiotensin receptor blockers
ACE: angiotensin-converting enzyme
DASH: Dietary Approaches to Stop Hypertension
LMWP: low-molecular-weight proteins
LMWC: Low molecular-weight chitosan
AQP1: aquaporin 1
PTECs: proximal tubule epithelial cells
PVD: poly (vinylpyrrolidone-co-dimethyl maleic acid)
PVP: polyvinylpyrrolidone
PEA: poly (ester amine)
TXNIP: thioredoxin-interacting protein
AuNPs: gold nanoparticles
ITSIONs: immunotargeted superparamagnetic iron oxide nanoparticles
UUO: unilateral ureteral obstruction
GLUT: Glucose Transporter
MNPs: Mesoscale NPs
PPE-AuNPs: pomegranate peel extract-stabilized gold nanoparticles
GPE: galactose polyethylenimine glycoprotein urethane: galactose polyethylenimine glycoprotein urethane
GECs: glomerular endothelial cells
FPs: foot processes
FcRn: Fc receptor
PCR: G protein-coupled receptor
